# Double transition metal MXene (Ti_x_Ta_4−x_C_3_) 2D materials as anodes for Li-ion batteries

**DOI:** 10.1038/s41598-020-79991-8

**Published:** 2021-01-12

**Authors:** Ravuri Syamsai, Jassiel R. Rodriguez, Vilas G. Pol, Quyet Van Le, Khalid Mujasam Batoo, Syed Farooq Adil, Saravanan Pandiaraj, M. R. Muthumareeswaran, Emad H. Raslan, Andrews Nirmala Grace

**Affiliations:** 1 Centre for Nanotechnology Research, Vellore Institute of Technology, Vellore, Tamil Nadu 632 014 India; 2 Davidson School of Chemical Engineering, Purdue University, West Lafayette, IN 47907 USA; 3 Institute of Research and Development, Duy Tan University, Da Nang, 550000 Vietnam; 4 King Abdullah Institute for Nanotechnology, King Saud University, P.O. Box 2455, Riyadh, 11451 Saudi Arabia; 5 Department of Chemistry, College of Science, King Saud University, PO Box 2455, Riyadh, 11451 Saudi Arabia; 6 Department of Self Development Skills, CFY Deanship, King Saud University, Riyadh, Saudi Arabia; 7 Department of Physics, College of Science, King Saud University, PO Box 2455, Riyadh, 11451 Saudi Arabia

**Keywords:** Batteries, Two-dimensional materials

## Abstract

A bi-metallic titanium–tantalum carbide MXene, Ti_x_Ta_(4−x)_C_3_ is successfully prepared via etching of Al atoms from parent Ti_x_Ta_(4−x)_AlC_3_ MAX phase for the first time. X-ray diffractometer and Raman spectroscopic analysis proved the crystalline phase evolution from the MAX phase to the lamellar MXene arrangements. Also, the X-ray photoelectron spectroscopy (XPS) study confirmed that the synthesized MXene is free from Al after hydro fluoric acid (HF) etching process as well as partial oxidation of Ti and Ta. Moreover, the FE-SEM and TEM characterizations demonstrate the exfoliation process tailored by the Ti_x_Ta_(4−x)_C_3_ MXene after the Al atoms from its corresponding MAX Ti_x_Ta_(4−x)_AlC_3_ phase, promoting its structural delamination with an expanded interlayer d-spacing, which can allow an effective reversible Li-ion storage. The lamellar Ti_x_Ta_(4−x)_C_3_ MXene demonstrated a reversible specific discharge capacity of 459 mAhg^−1^ at an applied C-rate of 0.5 °C with a capacity retention of 97% over 200 cycles. An excellent electrochemical redox performance is attributed to the formation of a stable, promising bi-metallic MXene material, which stores Li-ions on the surface of its layers. Furthermore, the Ti_x_Ta_(4−x)_C_3_ MXene anode demonstrate a high rate capability as a result of its good electron and Li-ion transport, suggesting that it is a promising candidate as Li-ion anode material.

## Introduction

Lithium-ion batteries (LiBs) have boosted the technological advances for the last three decades, especially in mobile and transport applications as well as potential large-scale energy storage systems for electric grid applications. Extensive research and development have been dedicated to explore and improve the key components of LIBs, including negative Li-ion host graphitic anodes^1^. Till 1980, attempts to make a rechargeable LIB failed due to the instability of the metallic lithium as anode material. The elemental lithium is the lightest metallic element with the lowest standard reduction potential being able to reach a high energy density. However, during its cycling, lithium metal loses its performance as Li dendrites grow^2^, which could puncture the separator and contact the cathode electrode, resulting in an electrical short circuit that leads to the battery failure or in the worst case causing its thermal runaway, also knows as “venting with flame”. The instability of the Li-metal led researchers to develop a non-metallic electrode for lithiation, fulfilling the demanded energy requirements^2–4^.

The development of exfoliated layered materials, such as graphene^5^, oxides^6,7^, and chalcogenides^8,9^, have attracted significant attention as Li-ion anode due to their remarkable electrochemical properties to store Li-ions, reversibly. Among the lamellar materials, the recent development of metal carbides and nitrides, known as MXenes, has become one of the most promising anode materials. MXene materials belong to a novel two-dimensional family, consisting of a thin-layered arrangement of transition metal carbides and carbonitrides^10^. Their novel physico-chemical properties have attracted a huge attention due to its applications in energy storage^11–14^, electronic^15–17^, catalysis^18–21^, sensor^18,22–24^, medicine^23–27^ and others from lab to industry^5,28^. The development of MXene materials can be summarized in three events: (i) the invention of MAX phases in 1970, discovered by Nowotny et al. as H-phases or M_2_BX^29^; (ii) the rebirth of MAX phases in 1993 by Barsoum et al.^30^ who first used the formula M_n+1_AX_n_ (n = 1–4) that later evolved to the term MAX phase where “ M ”is an early transition metal (Sc, Ti, V, Cr, Zr, Nb, Mo, Hf, Ta); A is group13 or 14 elements (Al, Si, P, S, Ga, Ge, As, Cd, Ln, Sn, Tl, Pb) and X is Carbon and/or Nitrogen^31,32^ (iii) MXene material, Ti_3_C_2_, was successfully prepared in 2011 by Barsoum et al*.*^33,34^ through the immersion and exfoliation processes of Ti_3_AlC_2_ (MAX phase) in hydrofluoric acid. As the acid treatment completely removed the aluminum (A element of MAX phase), forming an exfoliated 2D lamellar crystal similar to graphene, the suffix “ene” was added to the remaining MX phase, leading to the term MXene^33,34^.

Especially, Bi-metallic MXenes has attracted great attention because they present unique synergistic effects on their mechanical, textural and physicochemical properties^11, 16,35–39^. Titanium and tantalum alloys are well known for their unique combination and exceptional set of properties in the field of powder metallurgy, nuclear industry, reprocessing applications for storing nitric acid^40^ and biomedical applications^41–43^. Titanium carbide MXenes are the most reported material to date and tantalum carbide is gaining attention in the field of theranostics, cancer therapy and few other electrochemical applications. A combination of these two MXenes offers interesting properties and a unique set of chemistry. MXenes have an advantage in comparison with graphene in tunability of material properties. In the case of graphene, the only way to tune the material properties is by its functionalization, whereas in the case of MXenes, these can be done by alloying, processing and functionalization^44–47^. One more key factor governing the properties of the material is defects because they have a huge role in controlling conductivity^48^, mechanical strength and metallic or semiconductor behaviors^47,49^. For example, single layer graphene with zero defects is known as a semi-metal, whereas inducing defects into its sheet generates a semiconductor behavior. Lattice imperfections are unavoidable in the case of solid-state physics and/or chemistry. Thermal defects in titanium have been recently reported^47,49^. Carbon vacancies are unavoidable in the MAX phases so as the MXene. Although defect-free MXenes are proving its potential in various applications, it is still unclear whether these defects would improve or deteriorate the material performance. When tantalum and titanium form an alloy compound, Ti atoms will occupy the outer layer, while Ta atoms will prefer the middle layer^50^. In this case, these defects become more interesting because of Ta, which has a self-oxidizing behaviour that passivates its surface, thereby providing new physicochemical properties.

MXene materials have gained great attention in battery applications for its extraordinary electrochemical properties to store Li-ions between its layered structure^51^ and thus these materials offer a chemically stable surface^51,52^. Moreover, MXene materials have been utilized as conductive support of conversion^53,54^ and alloying^55,56^ materials, which is due to its excellent mechanical and electronic properties^57^. Huang et al.^54^ have reported sandwich like Na_0.23_ TiO_2_ /Ti_3_C_2_ MXene composite. This unique sandwich morphology can relieve the strain of the electrode on cycling and deliver an enhanced carrier transport mechanism to prevent aggregation of active material. Xia et al.^58^ used an interfacial assembly strategy to assemble Si porous nanospheres on titanium carbide MXene sheets; improving the electrode’s electron transport and stability. The MXene surface groups enable strong interaction with Si porous nanoparticles and develop pseudocapacitive behaviour, which can be advantageous for Li-ion storage. The Si-MXene assembly delivered an 1154 mAh/g at 0.2 A/g with good cycling stability. Meng et al.^59^ have explored black phosphorous quantum dots with Ti_3_C_2_ MXene as battery electrodes with enhanced pseudocapacitive capability. This interface enables high electrical conductivity, relieving stress on cycling with enhanced charge adsorption, and efficient interfacial electron transfer. The MXene composite electrode delivered 910 mAh/g at 100 mA/g with long cycling stability over 2400 cycles. Here, we report the preparation of a lamellar bi-metallic titanium–tantalum carbide MXene, Ti_x_Ta_(4−x)_C_3_ (where x = 2), with expanded interlayer spacing, as promising Li-ion host material with high capacity, excellent rate capability and long-term cyclability. The raw Ti_x_Ta_(4−x)_C_3_ MXene was synthesized through an etching process with HF, in which Al atoms were extracted from Ti_x_Ta_(4−x)_AlC_3_ MAX phase (where x = 2), promoting its structure delamination concurrently. The layered Ti_x_Ta_(4−x)_ C_3_ MXene displayed an expanded interlayer d-spacing of 3.37 Å with a layer thickness of 0.325 nm, which allows reversible Li-ion storage between its laminates. Therefore, Ti_x_Ta_(4−x)_ C_3_ delivered a remarkable reversible high specific discharge capacity of about 459 mAhg^−1^ at an applied C-rate of 0.5 °C with a coulombic efficiency of around 99% after 200 cycles, as well as an excellent rate capability.

## Materials characterization

X-ray diffractograms were obtained using a Bruker D8 XRD operated in Bragg–Brentano geometry with fixed slits (at room temperature). The vibrational modes were recorded using a Raman spectrometer (Horiba Scientific) with a 532 nm green laser. Morphological pictograms were recorded using supra-55 FE-SEM (Carl Zeiss) and a G2-20 TWIN TEM (FEI-TECNAI), respectively. High-resolution TEM images were recorded at 200 kV accelerating voltage integrated with a Gatan Orius CCD camera. Elemental composition was analyzed using a MultiLab 2000 XPS spectrometer (Thermo Scientific). All the elemental spectra were referenced with respect to the carbon (C1s—284.6 eV). Thermogravimetric Analysis was carried out using TA Instruments SDTQ600.

### Electrochemical evaluation

The bi-metal Ti_x_Ta_(4−x)_C_3_ MXene nanoparticles’ electrochemical performance as Li-ion host material was analyzed using potentiostatic cyclic voltammetry and galvanostatic charge–discharge cycling as per our earlier reports^6,60,61^. First, Ti_x_Ta_(4−x)_C3 MXene powder was mixed with conductive additive (super P carbon) and binder (PVDF) with a mass ratio of 80:10:10 in solvent (NMP). The resultant anode slurry was uniformly coated on a Cu foil and dried in vacuum at 80 °C for 24 h. The resultant anode films was punched out (12 mm of diameter).The electrochemical testing was carried out using coin-type cells 2032 in half-cell configuration with a Li and Celgard 2500 disks as counter electrode and separator, respectively, and 1.0 M LiPF6 electrolyte (EC: DEC—1:1).

## Results and discussion

**Figure 1:**
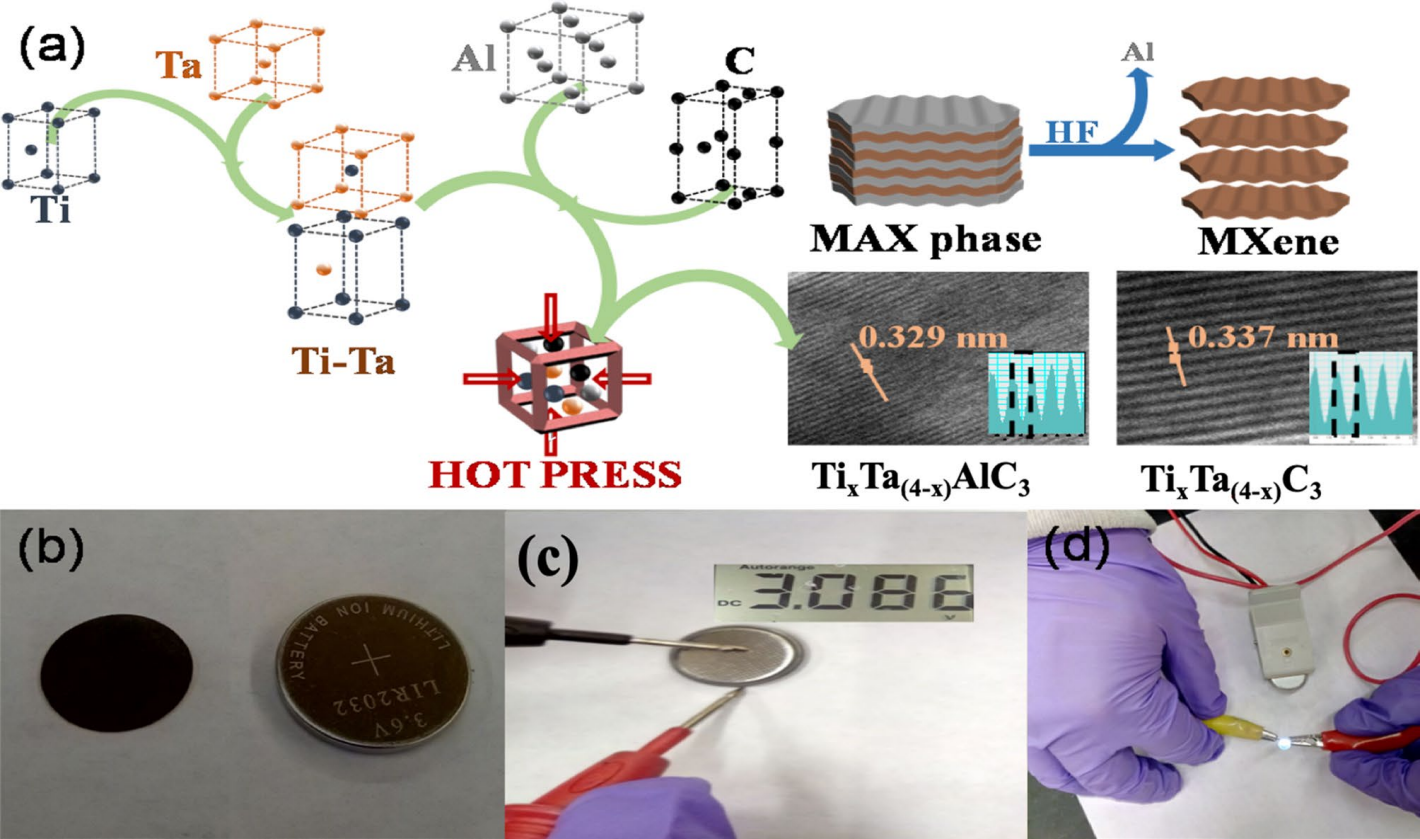
(**a**) A schematic illustration of the method followed to prepare bi-metallic Ti_x_Ta_(4−x)_C_3_ MXene, (**b**) MXene working electrode and its respective coin cell; tests of (**c**) open circuity voltage and (**d**) LED emitting white light using our MXene half-cell.

MXene compounds are traditionally prepared via a three-step process. First, a ball-milling is carried out to mix all the components efficiently. Then, a hot press treatment at a high temperature is performed to obtain a homogeneous MAX phase. Finally, an etching process with hydrofluoric acid is implemented to remove the “A” component of the MAX phase, giving a lamellar material, MXene. However, the preparation of the bi-metallic Ti_x_Ta_(4−x)_C_3_ MXene involves an extra-initial step that consists in the formation of a TiTa alloy through ball-milling as given in (Fig. 1a). The bi-metallic Ti_x_Ta_(4−x)_C_3_ MXene was used as active materials of the working electrode in a half-cell battery (Fig. 1b) that gives an open-circuit voltage (OCV) > 3.0 V vs. Li/Li^+^ after resting 24 h (Fig. 1c), which demonstrated its potential as anodic electrode turning on a white led (Fig. 1d).

**Figure 2:**
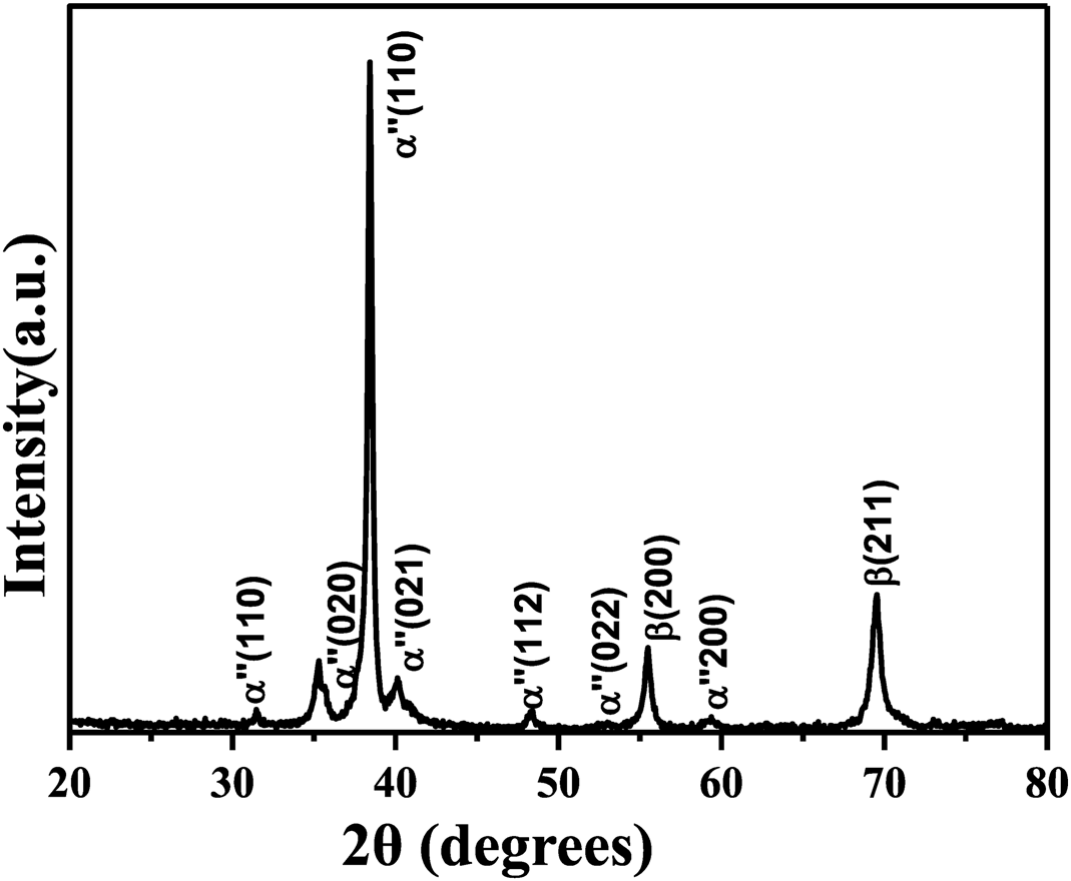
X-ray diffraction pattern of the titanium and tantalum alloy.

**Figure 3:**
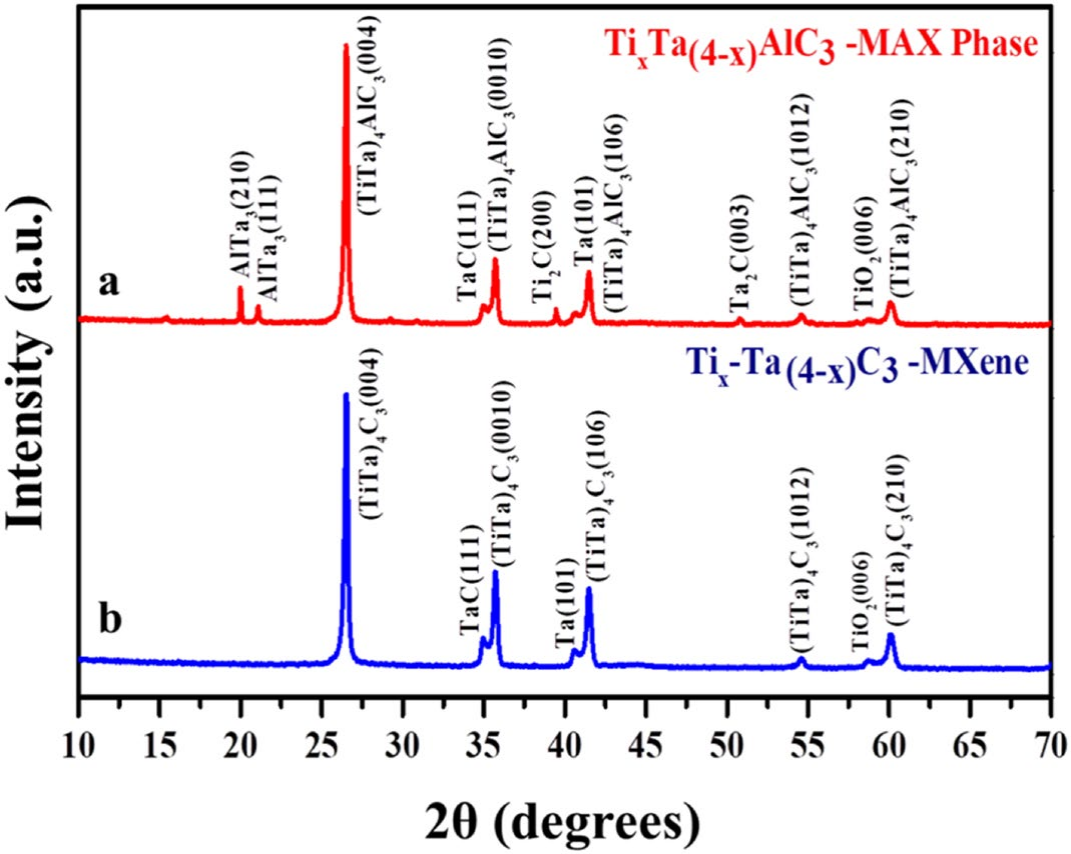
X-Ray Diffraction pattern of the synthesized bi-metallic (**a**) Ti_x_Ta_(4–x)_AlC_3_ MAX phase, red color; and (**b**) Ti_x_Ta_(4−x)_C_3_ MXene, blue color.

Figure 2 shows the XRD pattern of alloyed titanium and tantalum (TiTa), which were found out to be in the hexagonal phase matching with the JCPDS card number of 00-044-1294. The XRD pattern of the TiTa alloys, are narrow/sharp confirming micron sized particles. The major peaks in the system are martensitic α″ and minor being β (austenite phase, JCPDS card number of 00-044-1288), where α″ is inversely proportional to the particle size and β is directly proportional i.e. the particles with a smaller size, the XRD pattern will have dominated martensitic α″ peaks and minor β peaks^62^. In our case, there is also a major α″ for the alloyed TiTa. The powder XRD pattern of the Ti_x_Ta_(4−x)_AlC_3_ MAX phase is shown in Fig. 3. The XRD peaks of the synthesized sample are mainly ascribed to tantalum-titanium aluminum carbide with small traces of AlTa_3_, Ta_2_O, and TiO_2._ The obtained XRD pattern was in phase with the earlier reports matching with the tantalum aluminium carbide (ICSD-156383)^27,32^. A shift in 2θ was also observed because of the double ordered metal atoms. The synthesised samples were in hexagonal crystal system, which can also be seen in SAED pattern obtained from the TEM analysis as given in Fig. S3. Furthermore, by alloying Ti with Ta, the elemental Ti has a lower melting point than the Ta but as the particle is in micron size, they can be sintered together. In case of lower temperatures, Ti and Ta rich zones can appear in the material, but as MAX phases require a high temperature of 1500 °C, at this condition an interdiffusion of Ti and Ta atoms occurs at their interphase due to Kirkendall effect^63,64^. As there is a difference between Ti and Ta diffusion coefficients, there is an improper transport of atoms from one side of the interphase to the other, resulting in a partial diffusion and the formation of defects as vacancies or porosity in the zone, where the atoms move out^64^. Consequently, there is a downshift of the XRD peaks in the case of the MXene phase in comparison with its parental MAX, as a result of an increase in the C lattice parameter from after HF etching^32,39,65^. The minor reflections between 5° and 25° disappeared in the case of MXene phase because of the non-standard orientation of the sample (vertical orientation assuming based on the experimental data by Ghidiu et al.^66^). Also, the contamination peaks^66^ ascribed to AlTa_3_ and Ta_2_O are not observed as they are removed during the etching and washing processes.

The Raman spectrum of the synthesized bi-metallic Ti_x_Ta_(4-x)_AlC_3_ MAX phase and its corresponding Al etched Ti_x_Ta_(4−x)_C_3_ MXene phase are given in (Fig. S1). The Raman active modes are given from ω_3_ to ω_10_ because of TiTa, Al, and C^32,67–69^. The modes ω_3_, ω_6_, ω_9_ are due to the intermediate layer element “Aluminum”, which are suppressed due to the exchange of Al with its lower atoms (O, F, OH) or its removal during the HF etching processes.

**Figure 4:**
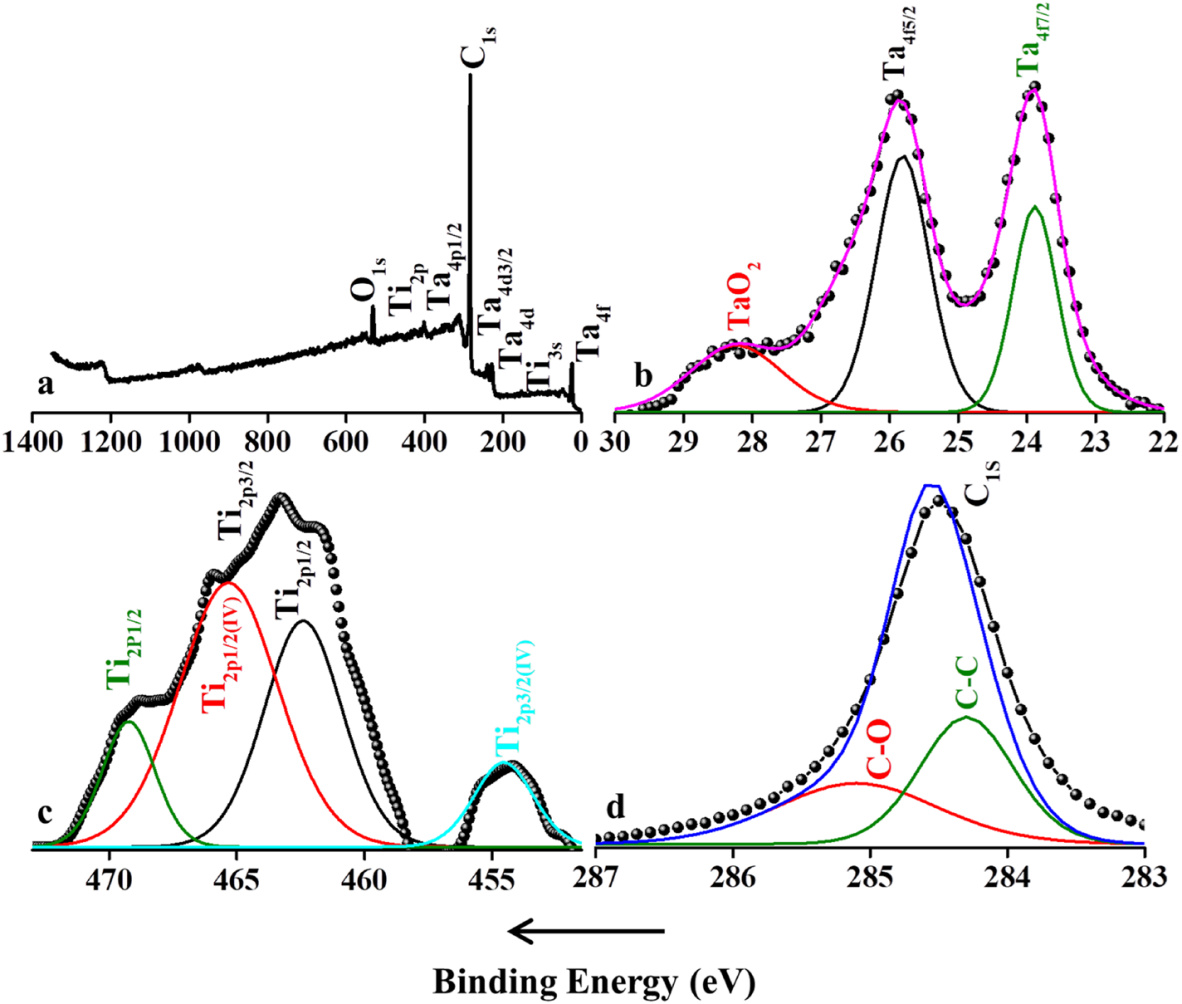
Full scan survey and deconvoluted XPS spectra of the synthesized bi-metal Ti_x_Ta_(4−x)_C_3_ (**a**) full scan survey of the synthesized MXene (**b**) deconvoluted spectrum of Ti, (**c**) deconvoluted spectrum of Ta, (**d**) deconvoluted spectrum of C.

The vibrational modes ω_4_ and ω_7_ have undergone a complete shift to lower wavenumbers due to Al etching as the lattice parameter “*c*” suffers an increment of its value. Also, there are few unidentified active modes in the spectrum, which could be assigned to the interaction of Ti and Ta atoms in the MXene phase and out-of-plane vibrations^70^. Furthermore, XPS elemental analysis was carried out to investigate the oxidation state and chemical composition of the synthesized MXene sample, which is given in (Fig. 4). Distinct peaks due to the presence of elements of titanium (Ti), tantalum (Ta), aluminum (Al) and oxygen (O) were visible in the full scan survey. The binding energies of tantalum and other elements are in phase with the existing reports^27,32,71^. The full scan spectrum of Ti_x_Ta_(4−x)_C_3_ MXene sheets in the Al region confirm that the synthesized materials were aluminum-free after HF etching processes (Fig. 4a). The spectrum of the Ta 4f. region was fitted by following 4f5/2 and 4f7/2 components that correspond to elemental Ta metals and its oxides as TaO_2_ (red) that are the two main species. The Ta 4f5/2 (black) and Ta 4f7/2 (green) binding energies are around 25.9 and 23.8 eV indicating that tantalum is in carbide environment (Fig. 4b). TaO_2_ has weak peaks probably that arise from the surface oxidation of the MXene sheets. The deconvoluted spectrum of Ti 2p spectra consists of four sets of doublet peaks corresponding to 2p3/2(IV) and 2p1/2(IV) of elemental metal and oxidized states of titanium (Fig. 4c). The dominant Ti 2p3/2 peaks at about 454.2 and 463.1 eV are assigned to the metallic Ti and Ti^4+^ states, respectively. In the de-convoluted spectrum of carbon, distant peaks at 284.6 eV, 283.3 eV and 283.7 eV are due to graphitic Sp^2^ carbon (Fig. 4d).

**Figure 5:**
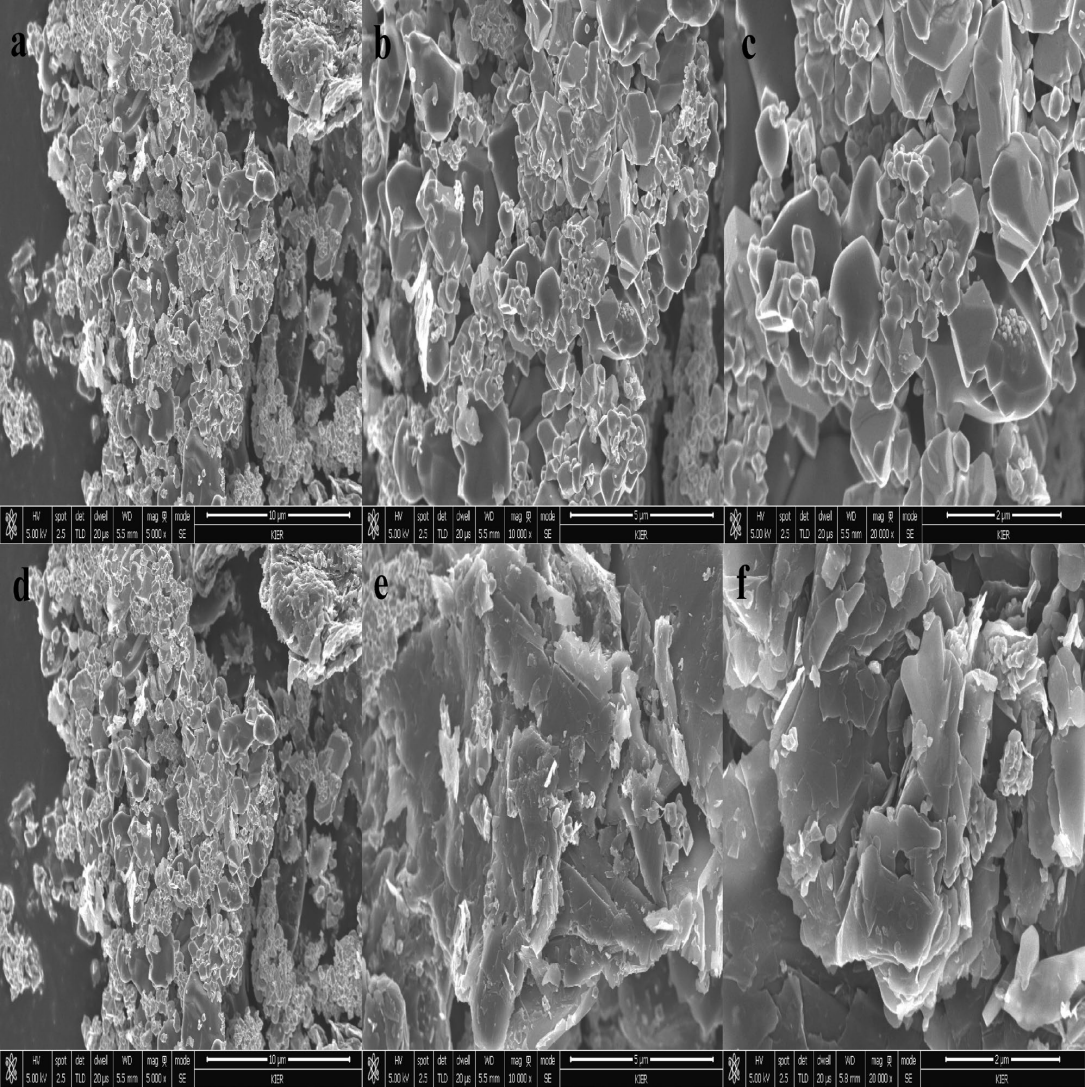
FE-SEM images of bi-metallic (**a**–**c**) Ti_x_Ta_(4−x)_AlC_3_ MAX phase and (**d**–**f**) Ti_x_Ta_(4−x)_C_3_ MXene at various magnifications.

**Figure 6:**
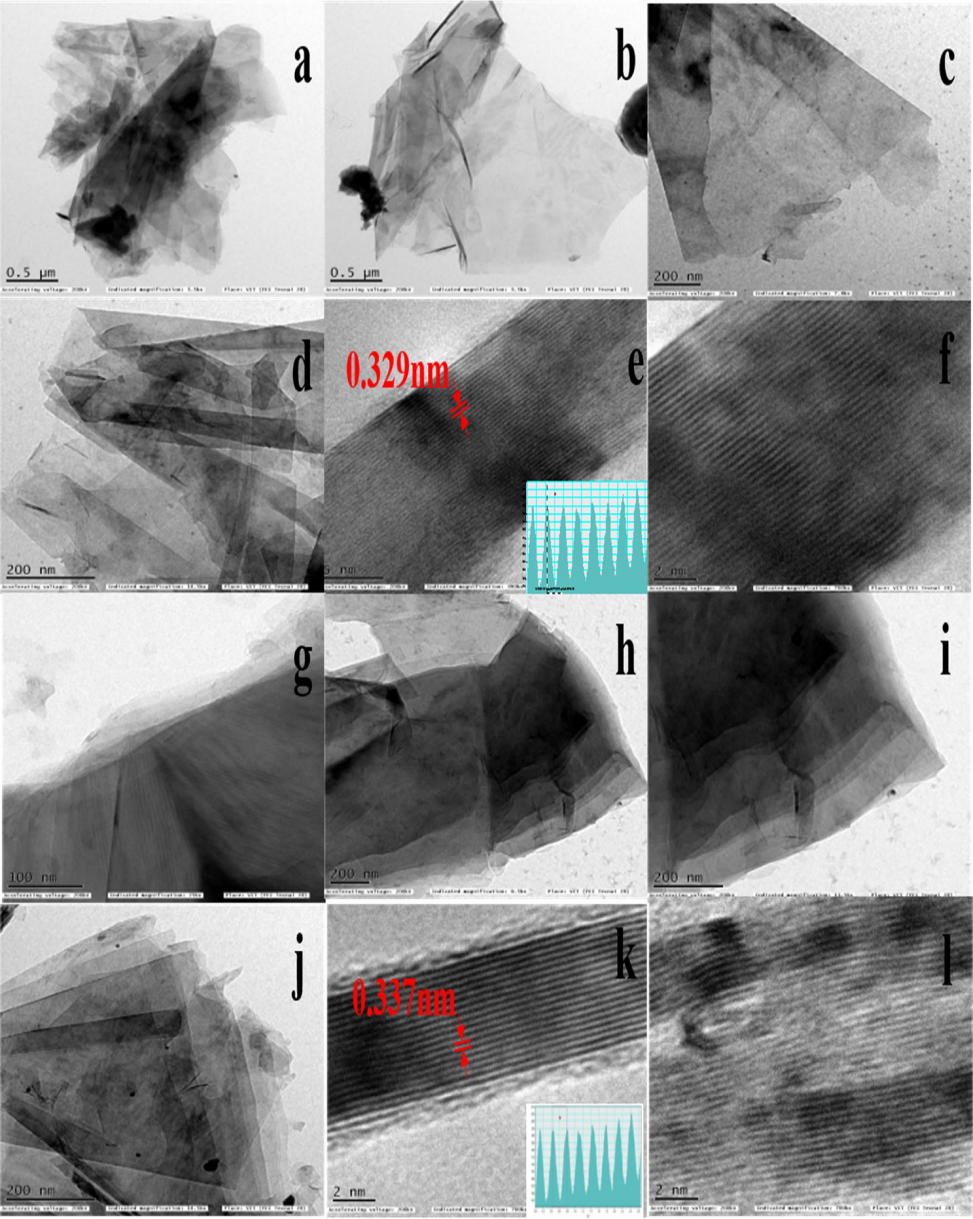
TEM micrographs of the bi-metallic Ti_x_Ta_(4−x)_AlC_3_ MAX phase (**a**–**f**) and Ti_x_Ta_(4−x)_C_3_ MXene (**g**–**l**).

FE-SEM images of the synthesized bi-metal Ti_x_Ta_(4−x)_AlC_3_ MAX phase and MXene samples are given in Fig. 5. The images show that the morphology of the synthesized MAX phase are in a layered solid structure (Fig. 5a–c). A small amount of nano layered structure was also observed, which can be attributed to the presence of other impurity phases in the sample. whereas the MXene sample shows that the layers are exfoliated during the etching processes (Fig. 5d–f). In case of both MAX phase and MXenes, at higher magnification of 10 µm both morphology looks similar. When the magnification is reduced to 5 µm and 2 µm, there is a clear difference in as the MAX phase with layered solid structure and etched MXene has an exfoliated layered structure with surface roughness because of etching processes. Furthermore, TEM micrographs of both the MAX phase and MXene are given in Fig. 6a–l, in which both the MAX phase and MXene exhibit layered structure and a thin arrangement enough to be electronically transparent; as the number of layers increases, this moved to a darker shade. Notably, the d-spacing of MXene increased from 0.329 nm to 0.337 nm at (004) plane due to the removal of Al atoms during the HF etching, which is in good agreement with that d-spacing calculated from the XRD data. As seen in (Fig. 6e,k), in comparision with the MAX phase, surface of the MXene sample is rough as a result of the surface functalization during thr etching processes. Understanding dislocations and kink bands were quite difficult until Morgiel et al*.* described them in their publication “Microstructure of Ti_3_SiC_2_ based ceramics in 1996”^72^. The dipole formed by the two dislocation segments is quite common in layered solids and other materials as MAX phases^73^ and mica^74^. It is evident from the FE-SEM and TEM images of both MAX phases and MXene that samples have a kink band (Fig. 5i). Since kinking is a type of buckling, porosity will enhance in the kinking effect^75,76^. The kink band favors electrochemical performance to a certain extent^77,78^. As seen in (Fig. 6f,i) in case of MAX phase, the HRTEM images were in darker shades because of thre layered solid behaviour, but it can be clearly distingushable in case of the MXene sample as there are different shades because of difference in thickness and spacing obtained due to removal of Al during the etchign processes.

**Figure 7:**
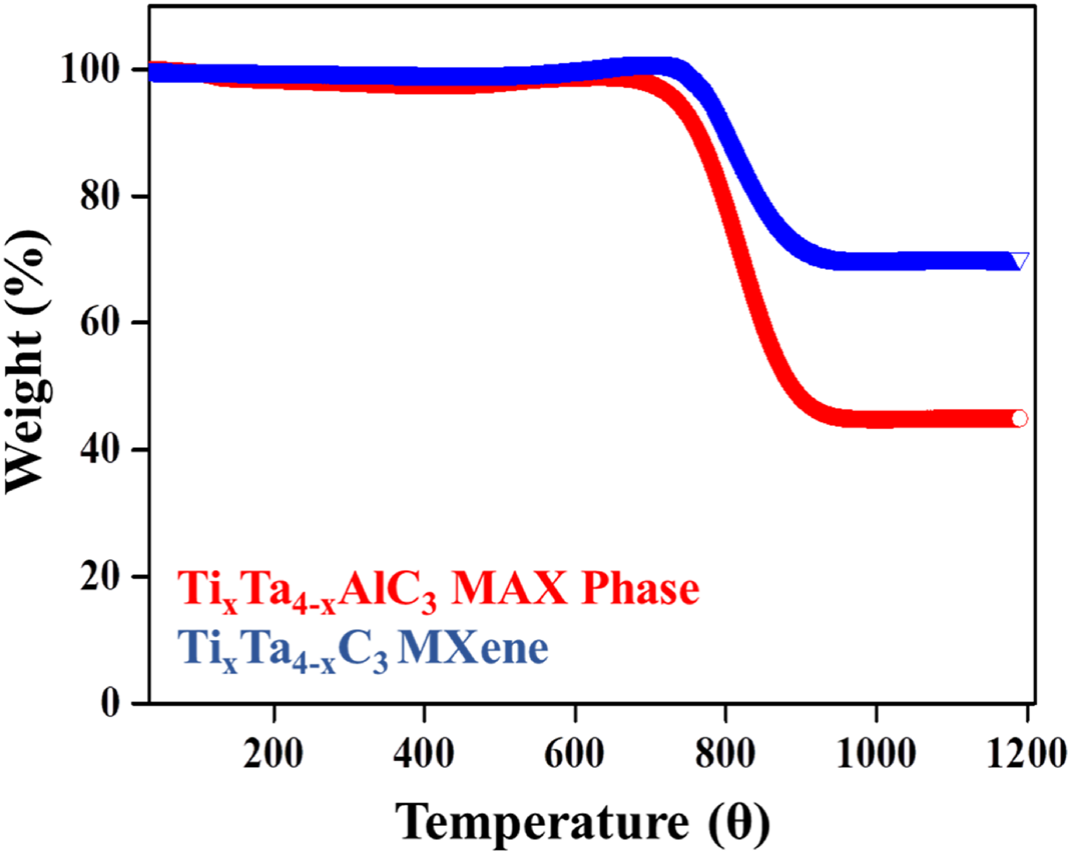
TGA behavior of the bi-metallic Ti_x_Ta_(4−x)_AlC_3_ MAX phase (Red) and Ti_x_Ta_(4−x)_C_3_ MXene (blue).

In order to understand the thermal behavior of the sample, thermogravimetric analysis was carried out from RT to 1200 °C. The analysis results are given in (Fig. 7), which shows a heavyweight loss for the MAX phase due to the loss of other impurities/mixed phases, whereas on the other hand in the case of MXene sample, the weight loss is less. In MAX phases, samples has the same behavior till 650 °C followed by huge weight loss from 650 to 900 °C, which might be because of TiO_2_ decomposition and/or due to formation of nanocrystals of TaC or TiC, which was also noticed in case of MXene sample and the weight loss patterns were in phase with the existing MXene literature^67,79–83^.

The lamellar bi-metallic Ti_x_Ta_(4−x)_C_3_ MXene was further evaluated using potentiostatic and galvanostatic methods to understand its electrochemical properties and redox mechanisms as Li-host anode material (Fig. S2) displays cyclic voltammograms recorded at scan rate of 0.2 mV s^−1^ from 0.01 to 3.0 V *vs* Li/Li^+^. During the initial cathodic cycle, irreversible broad peaks appear around 0.99 and 0.53 V *vs* Li/Li^+^^54^, which are related to the solid electrolyte interface (SEI) layer formation and Li-ion adsorption on the MXene pores^53^. The intercalation reaction between Ti_x_Ta_(4−x)_C_3_ and Li-ions occurs around 0.5 V *vs* Li/Li^+^, which can be expressed as: Ti_x_Ta_(4−x)_C_3_ + *x*Li^+^  + *x*e^−^  → Li_*x*_Ti_x_Ta_(4−x)_C_3_^53–55^. The subsequent CV profiles reveal a stable cathodic electrochemical process and suggest a two-step lithiation process as described in the previous literature^39^. During the anodic scans, a broad peak centered at 0.31 V *vs* Li/Li^+^ arises that is associated to Li-ion extraction process: Li_*x*_Ti_x_Ta_(4−x)_C_3_ → Ti_x_Ta_(4−x)_C_3_ + *x*Li^+^  + *x*e^−^^53–55^. Also, the constant charge capacity beyond 0.5 V vs. Li/Li^+^ can be ascribed to the reversible redox Li-ion desorption processes on the active material surface or pores.

**Figure 8:**
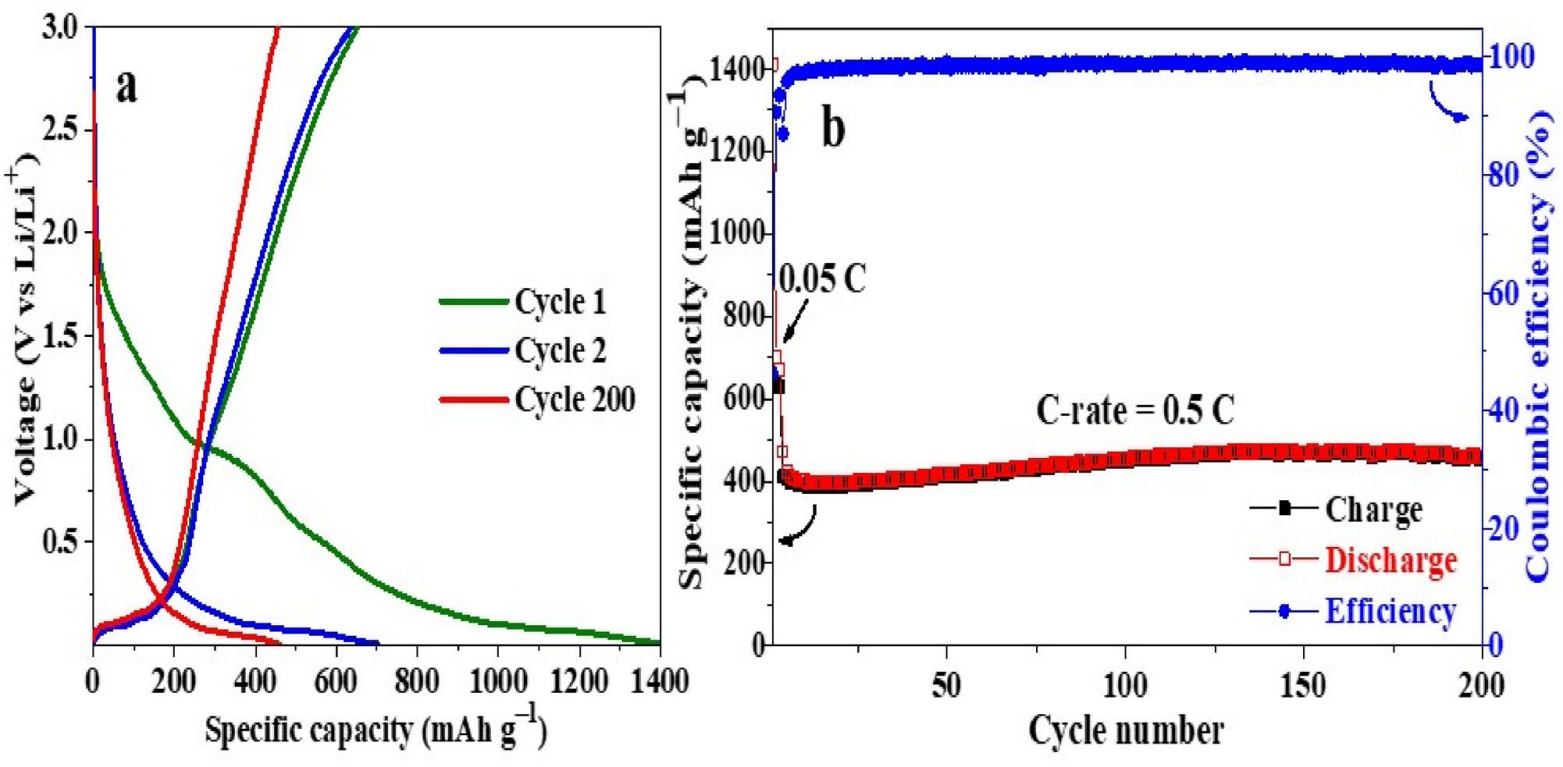
Electrochemical cycling performance of the MXene sample, Ti_x_Ta_(4−x)_C_3_: (**a**) (dis)charge profiles at cycle 1st, 2nd and 200th, (**b**) cycle performance after 200 cycles at C-rate of 0.5 °C.

The cyclability and rate capability of the layered Ti_x_Ta_(4−x)_C_3_ MXene were evaluated using galvanostatic (dis)charge measurements (Fig. 8a). The capacity delivered by the electrodes was estimated based on the mass of Ti_x_Ta_(4−x)_C_3_ and the applied C-rate was 1.0 °C = 372 mA g^−1^. Figure 7a shows the charge–discharge profiles of the Ti_x_Ta_(4−x)_C_3_ MXene anode for cycle 1st, 2nd and 200th recorded at a C-rate of 0.5 °C, between a voltage window from 0.01 to 3.0 V vs. Li/Li^+^. The Ti_x_Ta_(4−x)_C_3_ MXene anode gave an irreversible initial specific discharge capacity of around 1411 mAh g^−1^ at 0.05 °C-rate during the activation process. This non-reversible high value of discharge capacity was caused by the formation of the SEI layer that occurs during the 1st discharge cycle and might also be due to the formation of double Li layers between MXene layers^39^.

**Figure 9:**
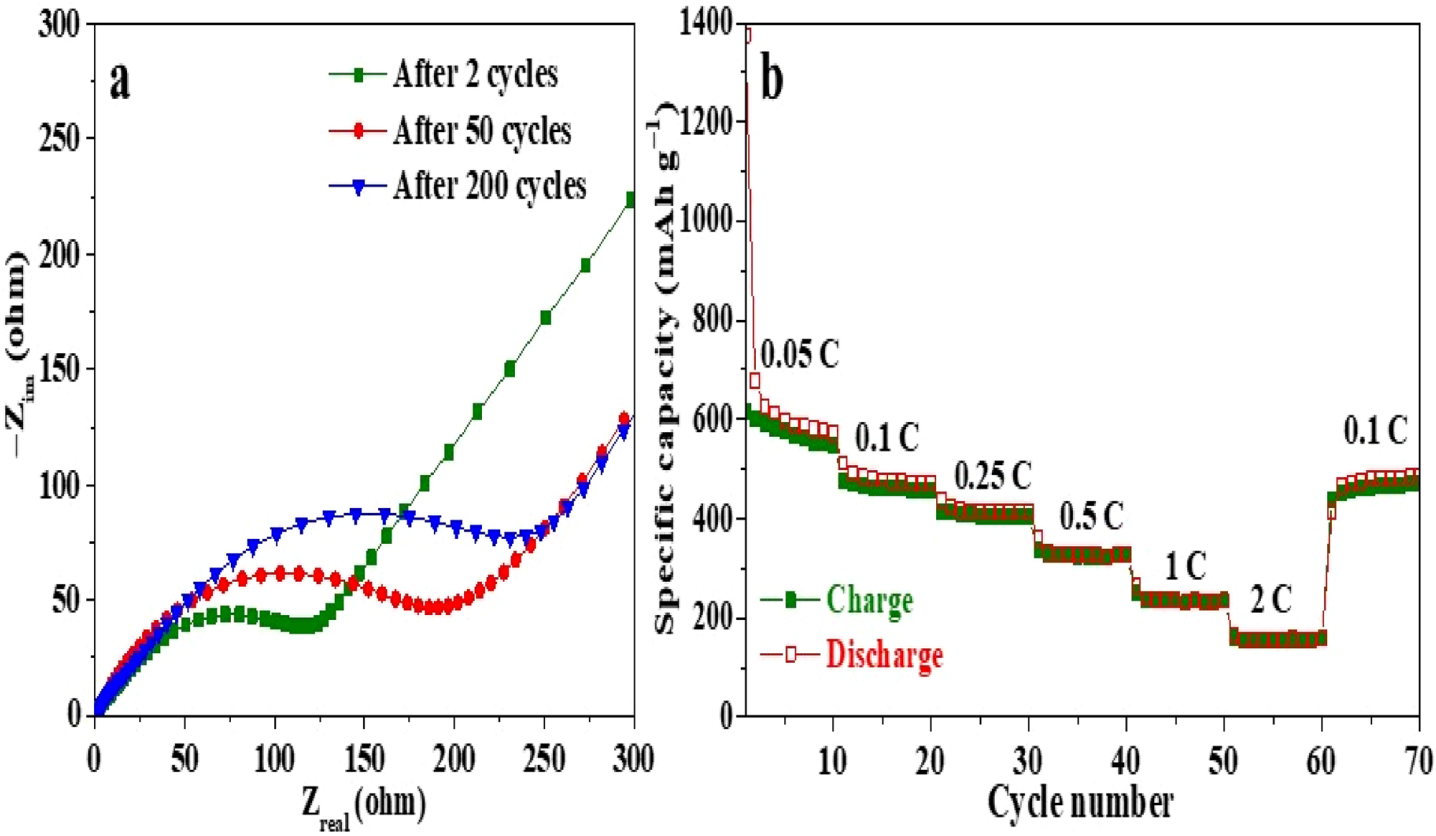
(**a**) EIS data before and after 200 cycles and (**b**) rate capability test.

Remarkably, the MXene anode delivers a high specific discharge capacity of about 459 mAh g^−1^ at a C-rate 0.5 °C with a capacity retention of about 97% after the activation process (cycle 3) and a coulombic efficiency of around 99% after 200 cycles. Figure 8b displays the cycle performance of the layered Ti_x_Ta_(4−x)_C_3_ MXene anode, which shows a stable cycling behavior with a slight increase of capacity during some cycles, reaching a discharge capacity of 476 mAh g^−1^ at a 0.5 °C-rate after 100 cycles, which decay until 459 mAh g^−1^ after 200 cycles. This loss of capacity could be associated with the disconnection of the active particles from the electrode as a result of the formation of a thick SEI between the MXene layers or might also be due to the restacking of the layered MXene material^84^, which can generate mechanical stress, cracks, and fractures inside the anodic electrode^85^. Figure 9a presents the Nyquist plot of fresh and cycled bi-metallic Ti_x_Ta_(4−x)_C_3_ MXene anode, in which the charge transfer resistance of the cycled electrode increases during cycling, indicating the formation and growth of a thick SEI layer as the number of cycles increases.

The rate capability of the bi-metallic Ti_x_Ta_(4−x)_C_3_ MXene anode was further evaluated under various C-rates during 10 D-C cycles per applied C-rate (Fig. 9b). The MXene anode exhibits an average of specific discharge capacities of 602, 482, 418, 332, 237, 156 mAh g^−1^ and back to 472 mAh g^−1^ at applied C-rate of 0.05, 0.1, 0.2, 0.5, 1 °C, and backward to 0.1 °C, respectively. The MXene anode displays a remarkably high C-rate capability recovering 98% of the delivered capacity of 482 mAh g^−1^ at C-rate of 0.1 °C after being cycled at various C-rate conditions. The extraordinary Li-ion storage of the bi-metallic Ti_x_Ta_(4−x)_C_3_ MXene anode could be related to the formation of a stable bi-metallic MXene material, which stores Li-ions on the surface of its layers during the redox process. The bi-metallic Ti_x_Ta_(4−x)_C_3_ MXene anode demonstrated good electronic connectivity between the active materials, carbon additive and binder, showing stable electrochemical performance, high capacity and good rate capability^51^. Results show that the synthesized MXene achieved an excellent electrochemical redox performance compared to the known MXenes (Table 1), which could be attributed to the formation of a stable, promising bi-metallic MXene material, which store Li-ions on the surface of its layers.

**Table 1:** Comparison of the bi-metallic Ti_x_Ta_(4−x)_C_3_ MXene performance with reported MXene anodes.

No	Electrode material	Battery type	Electrolyte salt	Cell voltage/V vs Li^+^/Li	Capacity/mAh g^−1^	Ref
1	Sn/Ti_3_C_2_	Li-ion	1 M LiPF_6_	1.0–3.0	635 at 0.5 °C	^86^
2	Ti_x_Ta_(4−x)_C_3_	Li-ion	1 M LiPF_6_	0.2–3.0	459 at 0.5 °C	This work
3	Nb_2_C/CNT	Li-ion	1 M LiPF_6_	1.0–3.0	420 at 0.5 °C	^87^
4	Nb_4_C_3_	Li-ion	1 M LiPF_6_	0.01–3.0	380 at 1 °C	^88^
5	V_2_C	Li-ion	1 M LiPF_6_	0.01–3.0	291 at 10 °C	^89^
6	V_2_C	Li-ion	1 M LiPF_6_	0.02–3.0	254 at 0.2 °C	^90^
7	MoS_2_/Ti_3_C_2_	Li-ion	1 M LiPF_6_	0.01–3.0	246.1 at 10 °C	^91^
8	Ti_3_C_2_	Li-ion	1 M LiPF_6_	0.05–3.0	123.6 at 1 °C	^92^
9	Ti_3_C_2_T_x_	Mg^2^^+^/Li^+^	0.4 M LiCl	0.2–2.0	105 at 0.1 °C	^93^
10	LVO/Ti_3_C_2_T_x_	Li-ion	1 M LiPF_6_	0.01–3.0	71 at 5 °C	^94^
11	Ti_2_C	Li-ion	1 M LiPF_6_	0.0–3.0	65 at 10 °C	^95^

## Conclusions

In conclusion, this is the first report, where bi-metallic titanium-tantalum carbide MXene Ti_x_Ta_(4−x)_C_3_ was successfully prepared through a conventional etching process of TixTa_(4−x)_AlC_3_ MAX phase. XRD and Raman analysis proved that the MAX phase was successfully prepared via a consecutive ball milling and heat treatment route. The lamellar MXene structural arrangement remains after the HF etching process, which is Al free according to the XPS analysis. FE-SEM and TEM characterizations exposed the exfoliated nature of the Ti_x_Ta_(4−x)_C_3_ MXene after removing the Al atoms from its corresponding MAX Ti_x_Ta_(4−x)_AlC_3_ phase. It also revealed a delaminated MXene structure with an expanded interlayer *d*-spacing of 3.37 Å. The lamellar Ti_x_Ta_(4-x)_C_3_ MXene was used as Li-ion host anode, giving a remarkably high reversible high specific discharge capacity of 459 mAh g^−1^ at of 0.5 °C rate with a coulombic efficiency of around 99% after 200 cycles and a capacity retention of about 97%. Also, the MXene anode presented a high rate capability, recovering 98% of the delivered capacity of 482 mAh g^−1^ at a C-rate of 0.1 °C after being cycled at various C-rate conditions. This new, electrochemically active Ti_x_Ta_(4−x)_C_3_ MXene provides a new approach to design high-capacity and stable Li-host anode materials for the next generation of rechargeable batteries.

## Experimental part

Titanium (Ti), tantalum (Ta), aluminum (Al), graphite (C) and hydrofluoric acid (HF) with high purity were acquired from Alfa Aesar, while toluene was purchased from Sigma-Aldrich. All the chemicals were used without any further purification. Also, double distilled water was used to wash the resultant MXene material.

### TiTa alloy preparation

The titanium-tantalum alloy (hereby referred to as TiTa) was synthesized by the powder metallurgy method using high purity Ti and Ta elements. During the alloy preparation stage, both elements were mixed with an atomic ratio of 1:1 through a ball milling process for 12 h at 250 rpm with a charge ratio of 1:10 under the presence of toluene. Then, the resultant sample was centrifuged and dried at 80 °C for 24 h, obtaining the TiTa alloy powder.

#### Synthesis of the MAX phase

To obtain Ti_x_Ta_(4−x)_AlC_3_ MAX phase, the pre-alloyed TiTa powder, aluminum and graphite in the proper mass amount were ball milled for 10 h with a charge ratio of 10:1. Then, the resultant mixture was loaded on to a graphite punching die and hot pressed at 1500 °C during 3 h under 1 Ton of pressure and a vacuum of 2 × 10^−5^ mbar.

#### Synthesis of the MXene

To convert the synthesized Ti_x_Ta_(4−x)_AlC_3_ MAX phase into Ti_x_Ta_(4−x)_C_3_ MXene, 1 g of the raw material was immersed in 20 mL of 40 v/v% HF solution and stirred for 4 days. The resultant dispersion was washed with double distilled water, vacuum filtered and dried at 80 °C for 24 h, obtaining the Ti_x_Ta_(4−x)_C_3_ MXene.

## Supplementary Information

Supplementary Information.
