# Author Correction: Double transition metal MXene (Ti_x_Ta_4−x_C_3_) 2D materials as anodes for Li-ion batteries

**DOI:** 10.1038/s41598-021-92167-2

**Published:** 2021-06-14

**Authors:** Ravuri Syamsai, Jassiel R. Rodriguez, Vilas G. Pol, Quyet Van Le, Khalid Mujasam Batoo, Syed Farooq Adil, Saravanan Pandiaraj, M. R. Muthumareeswaran, Emad H. Raslan, Andrews Nirmala Grace

**Affiliations:** 1 Centre for Nanotechnology Research, Vellore Institute of Technology, Vellore, Tamil Nadu 632 014 India; 2 Davidson School of Chemical Engineering, Purdue University, West Lafayette, IN 47907 USA; 3 Institute of Research and Development, Duy Tan University, Da Nang, 550000 Vietnam; 4 King Abdullah Institute for Nanotechnology, King Saud University, P.O. Box 2455, Riyadh, 11451 Saudi Arabia; 5 Department of Chemistry, College of Science, King Saud University, PO Box 2455, Riyadh, 11451 Saudi Arabia; 6 Department of Self Development Skills, CFY Deanship, King Saud University, Riyadh, Saudi Arabia; 7 Department of Physics, College of Science, King Saud University, PO Box 2455, Riyadh, 11451 Saudi Arabia

Correction to: *Scientific Reports* 10.1038/s41598-020-79991-8, published online 12 January 2021

The original version of this Article contained an error in the spelling of the author Syed Farooq Adil which was incorrectly given as Syed Adil Farooq. The original Article and accompanying Supplementary Information file have been corrected.

