# Supplementary material for: Double transition metal MXene (TixTa4−xC3) 2D materials as anodes for Li-ion batteries
Source: Sci Rep. 2021 Jan 12;11:688. doi: 10.1038/s41598-020-79991-8 (PMC7804453; doi:10.1038/s41598-020-79991-8)
Supplement: Supplementary file 1 — Supplementary Information. [file 41598_2020_79991_MOESM1_ESM.docx]

**Double Transition Metal MXene (Ti_x_Ta_4-x_C_3_) 2D Materials as Anodes****for Li-ion Batteries**

*Ravuri Syamsai,^1^ Jassiel R. Rodriguez,^2^ Vilas G. Pol,^2^ Quyet Van Le,^3^ Khalid Mujasam Batoo^4^, Syed Farooq Adil^5^, Saravanan Pandiaraj^6^, Muthumareeswaran MR^4^, Emad H. Raslan ^7^* and *Andrews Nirmala Grace^1,^**

^1^Centre for Nanotechnology Research, Vellore Institute of Technology, Vellore - 632 014, Tamil Nadu, India.

^2^Davidson School of Chemical Engineering, Purdue University, West Lafayette, IN 47907, USA.

^3^Institute of Research and Development, Duy Tan University, Da Nang 550000, Vietnam

^4^King Abdullah Institute for Nanotechnology, King Saud University, P.O. Box 2455, Riyadh-

11451, Saudi Arabia

^5^Department of Chemistry, College of Science, King Saud University, PO Box 2455, Riyadh-

11451, Saudi Arabia

^6^Department of Self Development Skills, CFY Deanship, King Saud University, Riyadh,

Saudi Arabia

^7^ Department of Physics, College of Science, King Saud University, PO Box 2455, Riyadh-

11451, Saudi Arabia

* Corresponding author: Andrews Nirmala Grace. E-mail: [anirmalagladys@gmail.com](mailto:anirmalagladys@gmail.com), anirmalagrace@vit.ac.in.

**
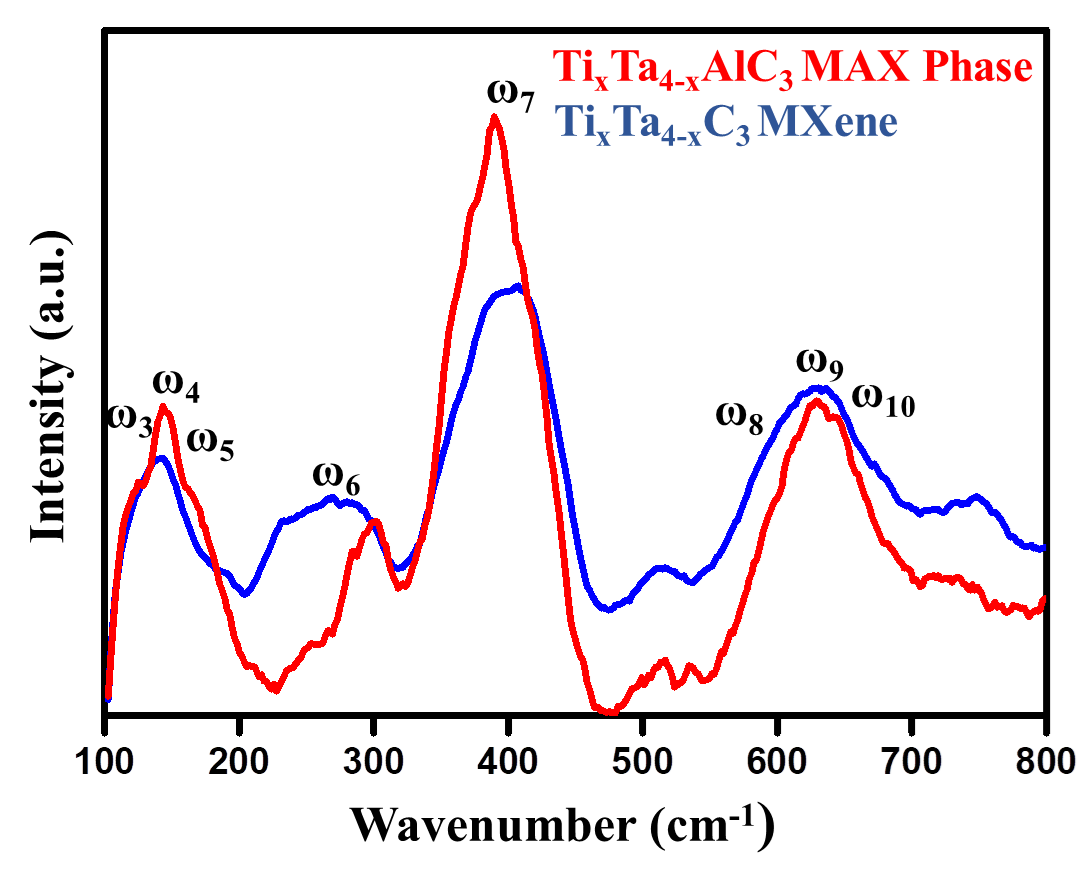
**

**Figure S1.** Raman spectrum of the synthesized bi-metallic Ti_x_Ta_(4-x)_AlC_3_ MAX phase and its corresponding Al etched Ti_x_Ta_(4-x)_C_3_ MXene phase.

**Figure S2.** Electrochemical performance of bi-metallic Ti_x_Ta_(4-x)_C_3_ MXene: cyclic voltammetry at 0.2 mV s^−1^.


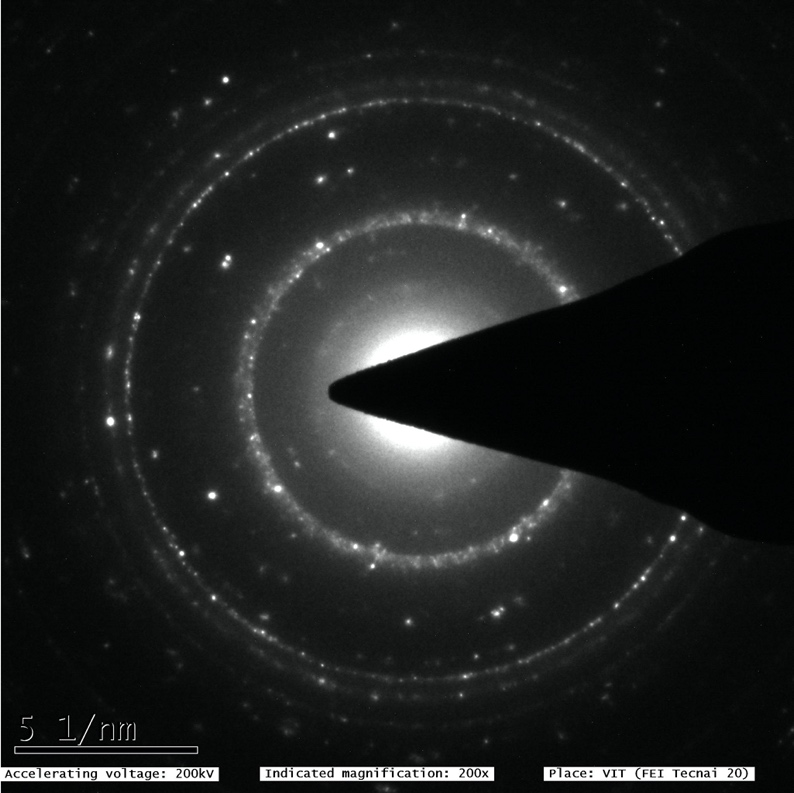


**Figure S3.** SAED pattern of Ti_x_Ta_4-x_C_3_ MXene.
